# Rapid decline in cardiac function in diabetic patients with calcified coronary artery disease undergoing hemodialysis: two case reports

**DOI:** 10.1186/s12872-021-02076-5

**Published:** 2021-06-03

**Authors:** Hironobu Yamaoka, Taira Yamamoto, Daisuke Endo, Akie Shimada, Satoshi Matsushita, Tohru Asai, Atsushi Amano

**Affiliations:** 1 Department of Cardiovascular surgery, Edogawa Hospital, Higashi Koiwa 2-24-18, Edogawa-ku, Tokyo, 133-0052 Japan; 2 Department of Cardiovascular Surgery, Juntendo University, 2-1-1, Bunkyo-ku, Tokyo, 113-8421 Japan

**Keywords:** Calcification, Coronary artery bypass grafting, Coronary artery disease, Hemodialysis, Diabetes mellitus

## Abstract

**Background:**

Clinical symptoms of patients on dialysis do not match the signs of coronary disease progression, making the prediction of the true progression of their medical condition in clinical settings difficult. Emergency and concomitant surgeries are significant risk factors of mortality following open-heart surgery in patients on hemodialysis.

**Case presentation:**

We report two cases of successful coronary artery bypass grafting (CABG) in patients on dialysis with a history of cardiac surgery. The first case describes a 65-year-old woman who had undergone aortic valve replacement 2 years ago and was hospitalized urgently, because of a sudden decline in heart function and hypotension. She had moderate mitral regurgitation with right ventricular pressure of 66 mmHg and poor left ventricular function [left ventricular ejection fraction (LVEF), 40%]. Cineangiography revealed an increase in the rate of stenosis in the left main trunk, from 25 to 99% at admission, in addition to 100% occlusion in proximal left anterior descending artery (LAD) and 99% stenosis in the proximal left circumflex artery (LCX). We inserted an intra-aortic balloon pump preoperatively and performed emergency surgery (Euro II risk score, 61.7%; Society of Thoracic Surgeons (STS) risk score, 56.3%). The second case described a 78-year-old man who had undergone surgery for left atrial myxoma 4 years ago and was hospitalized urgently due to dyspnea, chest discomfort, and an LVEF of 44% (Euro II risk score, 40.7%; STS risk score, 33.2%). Cineangiography revealed an increase in the rate of stenosis in the proximal LAD, from 25% (4 years ago) to 90% at admission, in addition to 99% stenosis in proximal LCX and 95% stenosis in the posterolateral branch of LCX. Both patients underwent emergency CABG due to unstable hemodynamics and decreased left ventricular function despite regular dialysis. The surgeries were successful, and the patients were discharged without any complications.

**Conclusions:**

In patients with multiple comorbidities and those who undergo dialysis treatment, calcified lesions in coronary arteries can progress severely and rapidly without any symptoms, including chest pain. Close outpatient management involving nephrologists and the cardiovascular team is necessary for patients on dialysis.

## Background

There are many reports on the coronary artery calcium (CAC) score and its associated factors in patients on hemodialysis. The CAC score has been estimated using the Agatston method with multidetector computed tomography (CT) [1]; rapid CAC progression has been associated with multiple risk factors, such as age, uremia, elevated C-reactive protein and phosphorus levels, calcium phosphate products, diabetes mellitus, duration of dialysis, hypertension, high triglyceride levels, and low high-density lipoprotein (HDL) cholesterol concentrations [2, 3]. Gürel et al. reported that the red blood cell distribution width is an independent predictor of the CAC score [4]. Another study suggested that activated platelets may induce leukocyte recruitment in the vascular walls and trigger inflammation, which is mainly observed in the pathogenic mechanisms underlying atherosclerosis [5]. However, in many patients on dialysis, the clinical symptoms do not match the progression of coronary disease, and predicting the true progress of the medical condition in clinical settings is difficult.

Cardiac surgeries in patients on hemodialysis have poorer operative outcomes than those in patients not on hemodialysis. In particular, emergency and concomitant surgeries are some of the significant risk factors of mortality following open-heart surgery in patients on hemodialysis [6, 7]. We, herein, present two reports of patients on hemodialysis with rapid worsening of calcified coronary artery disease and subsequent cardiac function.

## Case presentation

### Case 1

A 65-year-old woman presented with dyspnea on exertion. She had undergone aortic valve replacement (AVR) for aortic valve stenosis 2 years ago. The patient had a history of diabetes mellitus (on insulin lispro, 10-12-14-0 U; insulin detemir, 0-0-0-10 U per day) with onset at 55 years of age. Additionally, she had been undergoing hemodialysis since she was 58 years old, due to chronic renal failure with diabetic nephropathy. She also had hypothyroidism, secondary hyperparathyroidism, and sleep apnea; she never smoked. Her father had suffered from chronic renal failure and her mother had a malignant lymphoma. In this case, the inflammatory response decreased immediately after the first operation and remained low and stable, with C-reactive protein levels remaining between 0.1 and 0.5 mg/dL until the second operation.

The patient was admitted to our hospital because she noticed a decrease in her blood pressure during dialysis and dyspnea over the last 2 months with worsening of symptoms. On echocardiography, the left ventricular ejection fraction (LVEF) was found to have dropped sharply from 73 to 40% (New York Heart Association class III). Her height and weight were 151.8 cm and 63.3 kg, respectively. Her blood pressure was 88/50 mmHg, and her heart rate was 77 bpm (normal sinus rhythm). The laboratory data were as follows: hemoglobin, 14.3 g/dL; platelet count, 159 × 10^9^ /L; total protein, 6.9 mg/dL; albumin, 3.5 mg/dL; triglyceride, 105 mg/dL; low-density lipoprotein (LDL) cholesterol, 87 mg/dL; HDL-cholesterol, 32 mg/dL; serum-calcium (Ca), 9.6 mg/dL; serum phosphorus (P), 6.7 mg/dL; hemoglobin A1c (HbA1c), 5.2%; and brain natriuretic peptide (BNP), 2914 pg/mL.

**Fig. 1:**
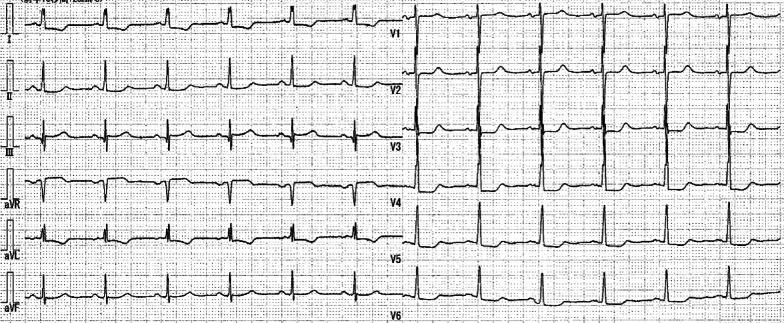
Preoperative electrocardiogram in Case 1. Electrocardiogram (ECG) showing normal sinus rhythm and ST depression in V4-V6

**Fig. 2:**
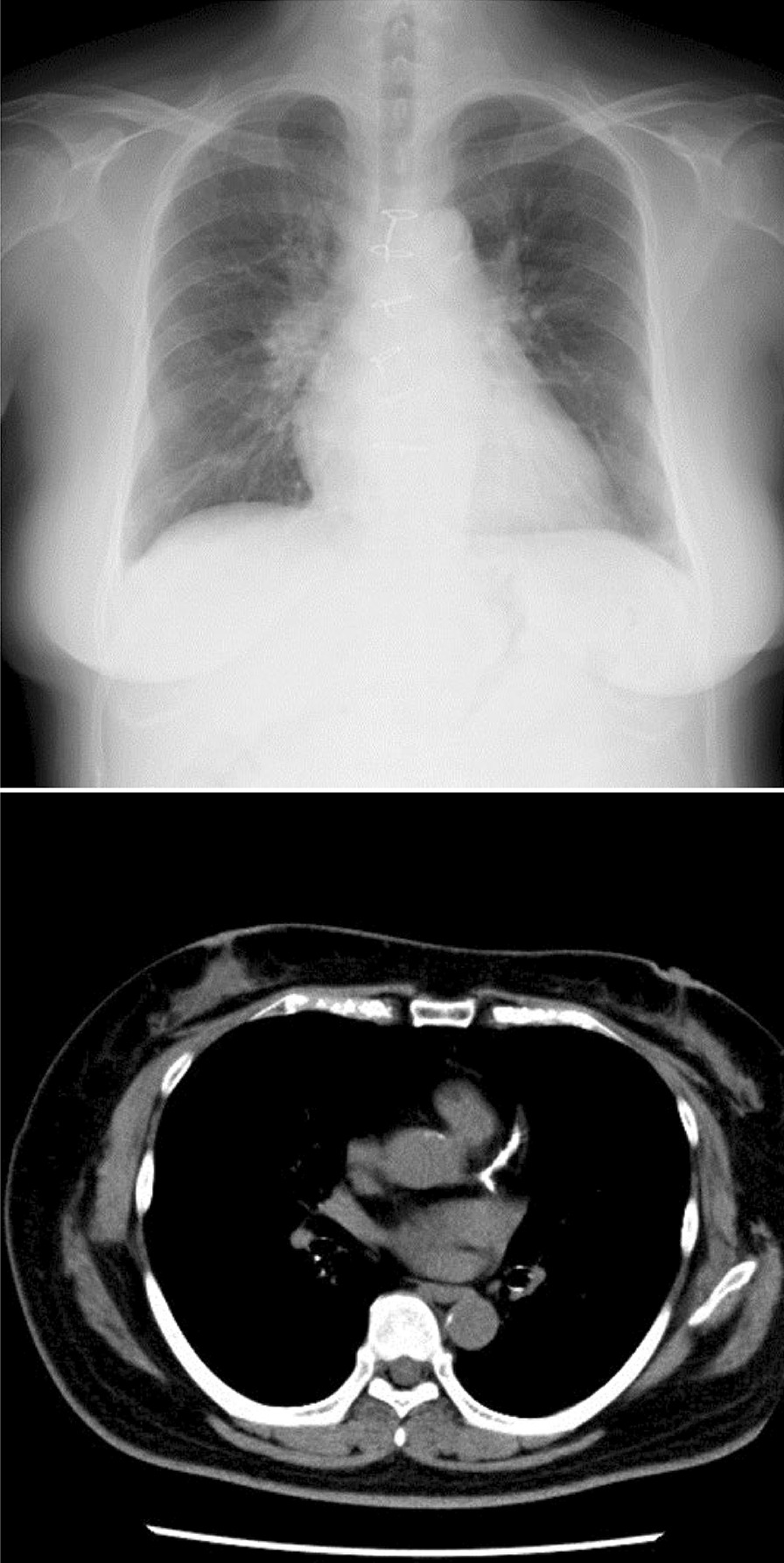
Preoperative chest radiography and computed tomography in Case 1. Chest radiograph showing a cardiothoracic ratio (CTR) of 57%, and computed tomography (CT) demonstrates a severely calcified coronary artery

Her oral medications included sucroferric oxyhydroxide (1500 mg/day), precipitated calcium carbonate (1500 mg/day), cinacalcet hydrochloride (12.5 mg/day), bisoprolol fumarate (2.5 mg/day), levothyroxine sodium hydrate (50 µg/day), linagliptin (5 mg/day), miglitol (100 mg/day), and pitavastatin calcium hydrate (2 mg/day). Electrocardiogram (ECG) revealed normal sinus rhythm and ST depression in V4-V6 (Fig. 1). Chest radiography revealed a cardiothoracic ratio (CTR) of 57%, and CT revealed a severely calcified coronary artery (Fig. 2). Ultrasonic echocardiography (UCG) after AVR revealed an effective orifice area of 1.2 cm^2^. She had moderate mitral regurgitation with a right ventricular pressure (RVP) of 66 mmHg. UCG indicated poor left ventricular function (left ventricular end-diastolic dimension (LVDd)/left ventricular internal dimension in systole: 48/38 mm, LVEF 40%) and hypokinesis in the anterior and posterior walls.

**Fig. 3:**
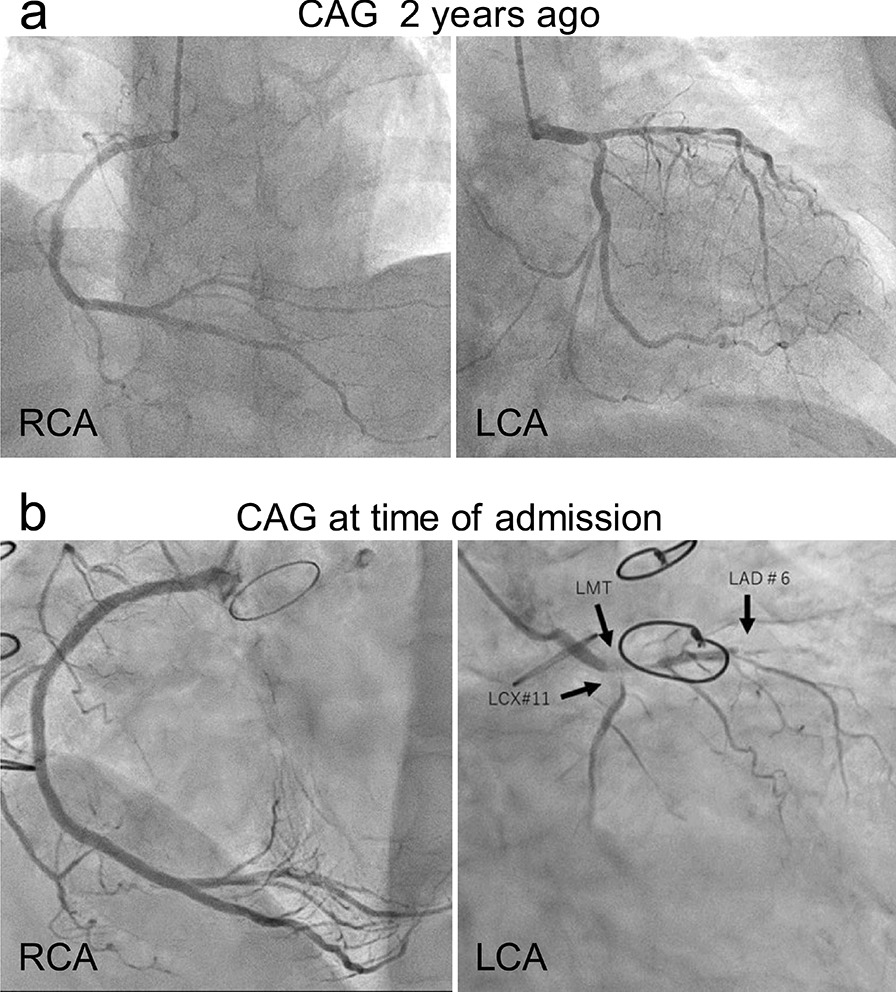
Coronary angiogram in Case 1. **a** Two years previously. No stenosis in the right coronary artery (RCA), 25% stenosis in the left main trunk (LMT), and 25% occlusion in the middle left anterior descending artery (LAD). **b** At admission. No stenosis in the RCA, 99% stenosis in the LMT, 100% occlusion in the proximal LAD, and 99% stenosis in the proximal left circumflex artery (LCX)

Two years previously, cineangiography had revealed 25% stenosis in the left main trunk (LMT) and the proximal left anterior descending artery (LAD); however, at admission, it revealed 99% stenosis in the LMT, 100% occlusion in the proximal LAD, and 99% stenosis in the proximal left circumflex artery (LCX) (Fig. 3a, b). During AVR, myocardial protection was provided using antegrade/retrograde perfusion, but not directly by selective perfusion in each coronary artery. We inserted an intra-aortic balloon pump (IABP) preoperatively and performed emergency surgery (Euro II risk score, 61.7%; STS risk score, 56.3%).

We performed sternotomy as was performed during AVR. After adhesion detachment, an artificial cardiopulmonary bypass (CPB) was established, with the inflow in the ascending aorta and the outflow in the right atrium. We performed coronary artery bypass grafting (CABG) (left internal thoracic artery [LITA] to LAD and saphenous vein graft to the left posterolateral branch [PL]), mitral annuloplasty (MAP) with a 26-mm CG Future (Medtronic plc, Dublin, Ireland), and tricuspid annuloplasty (TAP) with a 28-mm Contour 3D ring (Medtronic plc). The duration of overall surgery, CPB, and aortic cross-clamping was 393, 202, and 153 min, respectively. We removed IABP on the fourth postoperative day and postoperative ventilator management was completed on the fifth postoperative day. She stayed in the intensive care unit (ICU) for 13 days and was discharged on postoperative day 21. She was able to enjoy her daily routine life 1 year after the surgery, and her LVEF had improved from 40 to 55%.

### Case 2

A 78-year-old man presented with dyspnea on exertion. He had undergone resection of a left atrial myxoma 4 years prior to admission. He had a history of diabetes mellitus with onset at the age of 60; at the age of 71, he started undergoing dialysis for chronic renal failure due to diabetic nephropathy. He also had hypertension, hyperlipidemia, hypothyroidism, and secondary hyperparathyroidism, and was undergoing radiation therapy for prostate cancer. He had never smoked and his family history was unremarkable. He became aware of dyspnea on exertion in the previous 2 months, and was admitted to our hospital because his condition worsened. UCG revealed that the LVEF had decreased sharply from 73 to 44% (New York Heart Association class III). In this case, the inflammatory response remained low and stable at C-reactive protein levels of 0.3 to 0.5 mg/dL until the second operation.

His height and weight were 170.0 cm and 64.4 kg, respectively. His blood pressure was 86/48 mmHg and heart rate was 70 bpm (normal sinus rhythm). His laboratory data were as follows: hemoglobin, 10.2 g/dL; platelet count, 132 × 10^9^/L; total protein, 5.7 mg/dL; albumin, 3.3 mg/dL; triglycerides, 54 mg/dL; LDL-cholesterol, 44 mg/dL; HDL-cholesterol, 44 mg/dL; Ca, 8.1 mg/dL; P, 3.1 mg/dL; HbA1c, 7.0%; and BNP, 3461 pg/mL. His oral medications included aspirin (100 mg/day), furosemide (20 mg/day), carvedilol (2.5 mg/day), lansoprazole (15 mg/day), precipitated calcium carbonate (3000 mg/day), rosuvastatin calcium (5 mg/day), and nicorandil (15 mg/day).

**Fig. 4:**
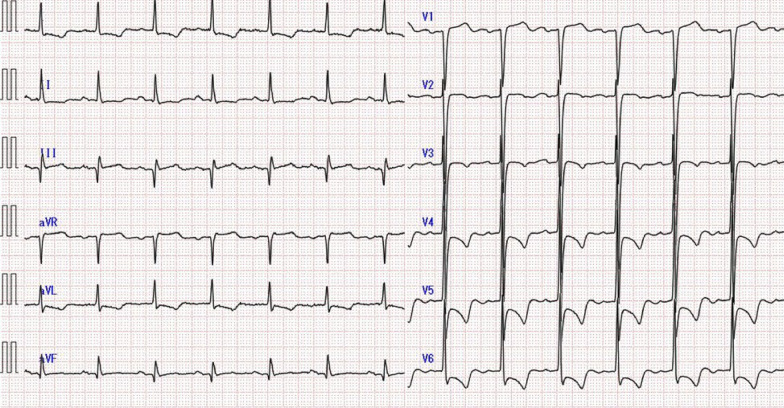
Preoperative electrocardiogram in Case 2. Electrocardiogram (ECG) showing normal sinus rhythm with Q waves in leads II, III, and aVF, and ST segment depression in V5-V6

**Fig. 5:**
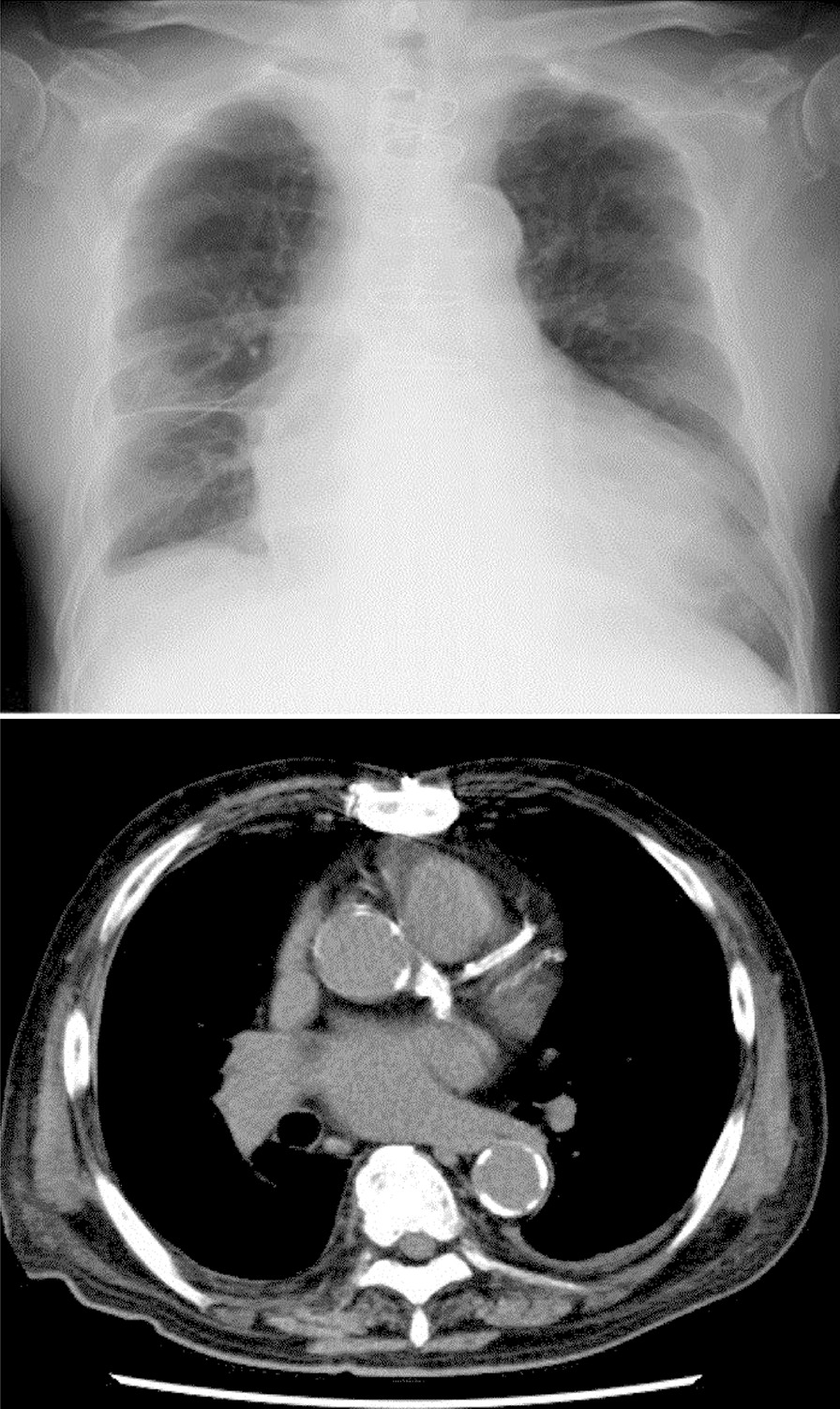
Preoperative chest radiography and computed tomography in Case 2. Chest radiography and computed tomography (CT) showing a CTR of 72% and severely calcified coronary arteries

**Fig. 6:**
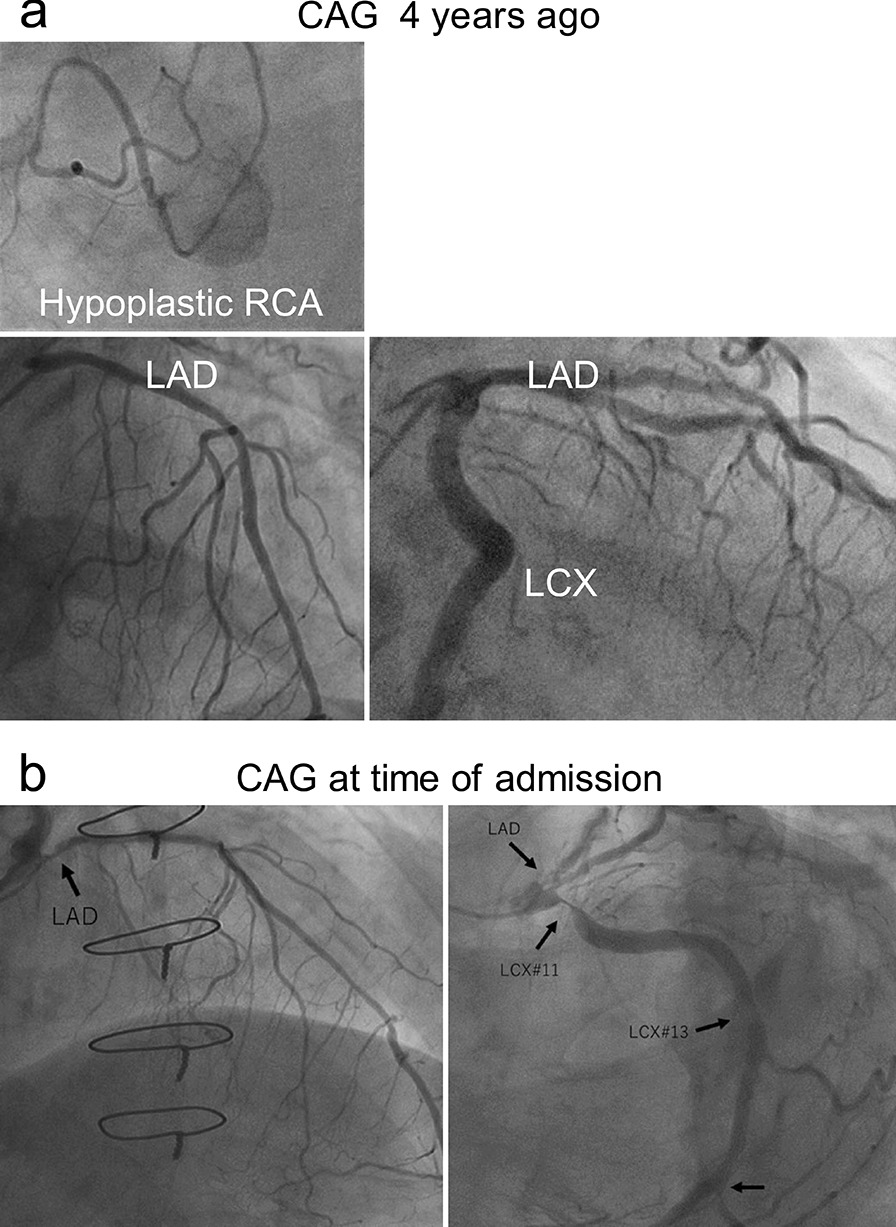
Coronary angiogram in Case 2. **a** Four years previously. A right coronary artery hypoplastic artery with no stenosis in the left anterior descending artery (LAD) and left circumflex artery (LCX). **b** At admission. The images show 90% stenosis in the proximal LAD, 99% stenosis in the proximal LCX, and 90% stenosis in the posterolateral branch of the LCX

ECG revealed normal sinus rhythm with Q waves in leads II, III, and aVF, and ST segment depression in V5-V6 (Fig. 4). Chest radiography and CT revealed a CTR of 72% and severe calcified coronary arteries (Fig. 5). UCG revealed poor left ventricular function (LVDd/Ds, 56/43 mm; LVEF, 44%; inferoposterior wall hypokinesis; and mild MR with RVP of 29 mmHg). Cineangiography 4 years ago had shown only 25% stenosis in the proximal LAD; at admission, it revealed 90% stenosis in the same proximal LAD, 99% stenosis in the proximal LCX, and 95% stenosis in the posterolateral branch (PL) of LCX (Fig. 6a, b). He had low blood pressure during dialysis and underwent emergency surgery (Euro II risk score 40.7%; STS risk score, 33.2%).

**Fig. 7:**
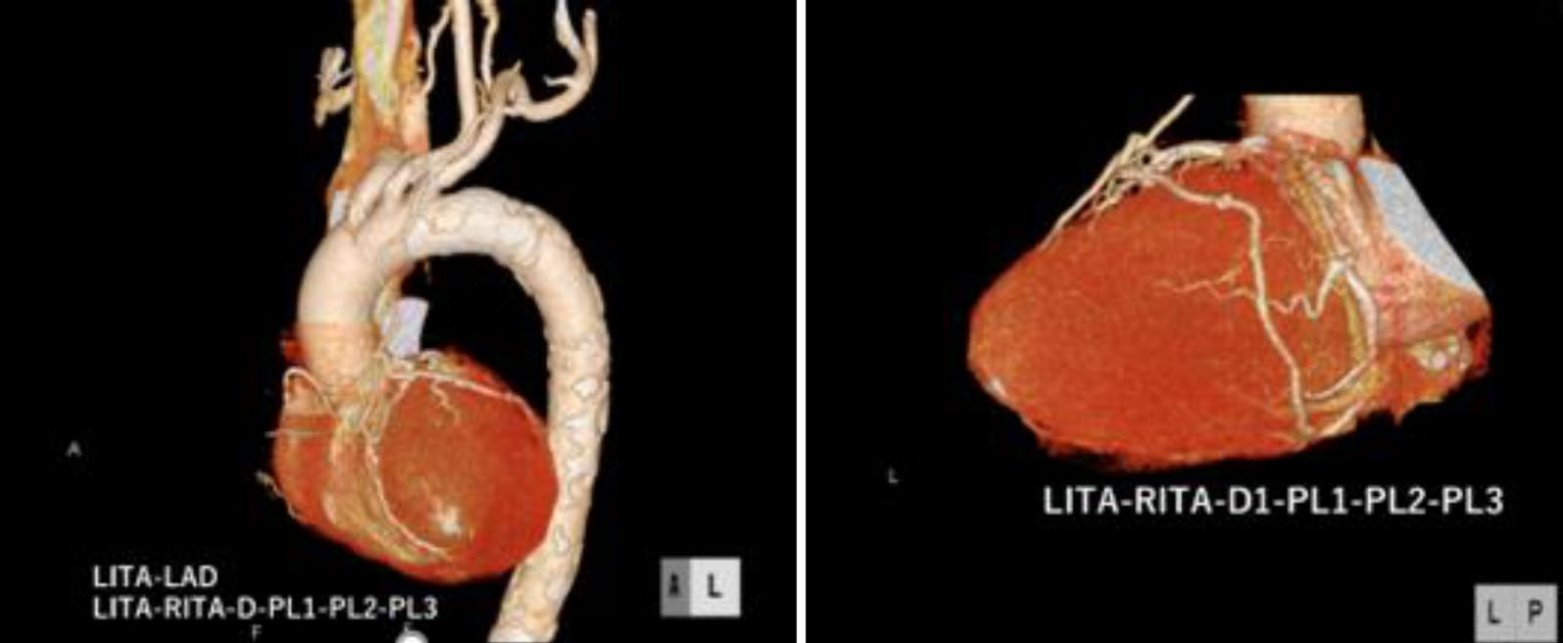
Postoperative three-dimensional computed tomography (3D-CT) in Case 2. All grafts are patent

We performed a sternotomy similar to the one performed previously, and then performed CABG (LITA-LAD, and LITA-right internal thoracic artery [right internal thoracic artery, RITA-Diagonal branch-PL1-PL-2-PL3]). The duration of surgery and CPB was 514 and 172 min, respectively. The postoperative ventilator management was completed in 9 h. He stayed in the postoperative ICU for 4 days and was discharged on postoperative day 10. The LVEF had improved from 44 to 50%, and all grafts were well patent (Fig. 7).

## Discussion and conclusions

In Japan, the number of patients who require hemodialysis is continuously increasing; 340,000 patients reportedly required hemodialysis in 2018. However, according to a Japan Society for Dialysis Therapy report, the 1-year crude death rate for patients on dialysis in Japan is approximately 9%, and the 1-year, 5-year, and 10-year survival rates are 89%, 60%, and 35%, respectively [8]. Approximately 50% of all deaths in patients on maintenance dialysis are due to heart diseases, such as heart failure and myocardial infarction. Coronary artery disease is one such leading cause and is strongly associated with adverse events. According to cardiac catheterization results in patients on maintenance dialysis, more than 60% of patients had ≥ 75% stenosis in their main coronary artery, and the average number of diseased coronary arteries per patient was 3.3 [9]. At least 35% of patients with chronic kidney disease have myocardial ischemia or angina when referred to a nephrologist [10].

End-stage renal disease is mainly due to calcification of the arterial intima and media. Intimal calcification is observed in coronary arteries, carotid arteries, and the aorta, and is associated with hyperlipidemia, hypercholesterolemia, and atherosclerosis due to diabetes. Medial calcification, also known as Monckeberg’s sclerosis, is a hallmark sign in patients with chronic kidney disease; it is primarily caused by hyperphosphatemia and is observed in muscular arteries, including the tibial and femoral arteries [11]. Fibroblast growth factor has also been reported to be associated with aortic and peripheral vascular calcification through changes in minerals and parathyroid hormones related to chronic kidney disease [12]. In addition to coronary stenosis, a mismatch between oxygen supply and demand may cause secondary ischemia in patients on maintenance dialysis. Reduced aortic compliance due to atherosclerosis leads to increased pulse wave velocity [13], thereby causing systolic hypertension and left ventricular hypertrophy [14].

Zoccali et al. reported that multiple Cox regression analysis, which included age, diabetes, smoking, and serum homocysteine levels showed that a 1 g/m ^2.7^/month increase in left ventricular mass index was associated with a 62% increase in cardiovascular events [15]. As chronic kidney disease progresses, the prevalence of left ventricular hypertrophy also increases. Additionally, reduced erythropoietin secretion causes chronic anemia, which ultimately leads to reduced oxygen supply. In patients with end-stage renal disease, symptoms of decreased cardiac function and heart failure may precede coronary stenosis. The prevalence of diabetes is high in these patients, and consequently, the early signs of cardiac ischemia are often undiagnosed secondary to autonomic damage due to diabetic neuropathy. Furthermore, despite anemia, patients on maintenance dialysis usually remain asymptomatic due to blood pressure regulation and volume adjustment by dialysis. This may worsen the symptoms of myocardial ischemia irrespective of the progression of arteriosclerosis. Using the Holter monitor, a study showed asymptomatic ischemia in 40% of patients on maintenance dialysis [16]. Additionally, outpatient clinics limit the use of contrast media in patients with dialysis and renal dysfunction; this can result in delayed coronary artery examination and assessment of the true severity of the disease [17].

Cardiac function may have declined due to many factors, and not only hemodialysis. However, we believed that it was an essential factor in the decline of cardiac function due to the progression of myocardial ischemia, owing to aggravation of coronary artery stenosis. Cardiac function was improving with the improvement of ischemia after CABG in these two cases. We speculated that it was essential to perform regular examinations in these patients while always suspecting progression of myocardial ischemia.

The strengths of this case report include the fact that both patients were managed successfully and lived after their respective surgeries. We also found the probable cause for their illness, with ample support from the literature. The limitations to this case report are that the patients had been managed poorly before emergency intervention; this prevented us from taking preventive measures.

In conclusion, we successfully performed emergency CABG (and valve surgery) in two patients on hemodialysis with a history of prior cardiac surgery. The rapid response and cooperation with the Department of Cardiology permitted us to obtain good surgical results without complications. Many patients on maintenance dialysis have severe calcified coronary arteries; however, they rarely complain of symptoms of myocardial ischemia, such as angina. With the progression of coronary artery lesions, there is a sudden decline in cardiac function, and emergency surgery may be required. Therefore, in addition to nephrologists, cardiologists and cardiovascular surgeons should carefully monitor outpatients, and especially those on dialysis.

## Data Availability

Data associated with this manuscript are not publicly available, but can be made available by the corresponding author upon reasonable request.

## Abbreviations

AVR: Aortic valve replacement
BNP: Brain natriuretic peptide
CAC: Coronary artery calcium
CT: Computed tomography
CTR: Cardiothoracic ratio
ECG: Electrocardiogram
HDL: High-density lipoprotein
IABP: Intra-aortic balloon pump
LAD: Left anterior descending
LCX: Left circumflex artery
LDL: Low-density lipoprotein
LITA: Left internal thoracic artery
LMT: Left main trunk
LVDd: Left ventricular end-diastolic dimension
LVEF: Left ventricular ejection fraction
PL: Posterolateral
UCG: Ultrasonic echocardiography
RVP: Right ventricular pressure

## Declarations

### Patients’ perspectives

Both patients in the presented cases were satisfied with explanations of their treatment before surgery and the postoperative course and test results.
